# Optimization of Ultrasonic Extraction of Phenolic Antioxidants from Green Tea Using Response Surface Methodology

**DOI:** 10.3390/molecules181113530

**Published:** 2013-10-31

**Authors:** Lan-Sook Lee, Namhyouck Lee, Young Ho Kim, Chang-Ho Lee, Sang Pil Hong, Yeo-Won Jeon, Young-Eon Kim

**Affiliations:** Korea Food Research Institute, Seongnam, Kyonggi 463-746, Korea; E-Mails: sohee0809@hanmail.net (L.-S.L.); lnh@kfri.re.kr (N.L.); youngho@kfri.re.kr (Y.H.K.); chang@kfri.re.kr (C.-H.L.); sphong@kfri.re.kr (S.P.H.); 02callalily@naver.com (Y.-W.J.)

**Keywords:** green tea, caffeine, antioxidant, ultrasonic extraction, response surface methodology

## Abstract

Response surface methodology (RSM) has been used to optimize the extraction conditions of antioxidants with relatively low caffeine content from green tea by using ultrasonic extraction. The predicted optimal conditions for the highest antioxidant activity and minimum caffeine level were found at 19.7% ethanol, 26.4 min extraction time, and 24.0 °C extraction temperature. In the predicted optimal conditions, the experimental values were very close to the predicted values. Moreover, the ratio of (EGCg + ECg)/EGC was identified a major factor contributing to the antioxidant activity of green tea extracts. In this study, ultrasonic extraction showed that the ethanol concentration and extraction time used for antioxidant extraction could be remarkably reduced without a decrease in antioxidant activity compared to the conventional extraction conditions.

## 1. Introduction

Tea is the most widely consumed beverage in the World next to water and is known for its various health benefits [1,2]. Polyphenolic compounds, a complex group of secondary metabolite substances found in plants, act as antioxidants, UV protectants, antimutagenic, anticarcinogenic, and antimicrobial agents. Green tea contains various classes of polyphenols, and up to 90% of its polyphenols is composed of flavan-3-ols (tea catechins). (+)-Catechin (C), (−)-epicatechin (EC), (+)-gallocatechin (GC), (−)-epigallocatechin (EGC), (−)-epicatechin gallate (ECg), (−)-epigallocatechin gallate (EGCg), and (+)-gallocatechin gallate (GCg) are the major catechins found in green tea leaves [3]. Among these catechins, EGCg is well known as a strong antioxidant. Green tea extracts have been used in functional food products to enhance the health benefits and to increase the shelf life because of their high antioxidant activity. For example, the tea catechins have been added to cereals, cakes, biscuits, ice cream, instant noodles, fried snacks, sausages, and soft drinks [4,5]. Conversely, the caffeine component of green tea has both positive and negative effects on health. It is a central nervous system and metabolic stimulant, and reduces physical fatigue and drowsiness at low doses. However, at high doses (typically greater than 300 mg), caffeine stimulates the cerebral cortex to induce excitation in the central nervous system, and also causes irritation of the gastrointestinal tract and sleeplessness for certain people [6,7]. Thus, it is necessary to reduce the contents of caffeine in green tea extracts to realize its optimum health benefits.

In order to extract the green tea, hot water and organic solvent extraction are the most commonly used procedures [8,9,10,11,12]. In general, the yield of solvent extraction depends on the type of solvents, with varying polarities, extraction time, and temperature, as well as on the physicochemical properties of the samples. Therefore, there is no single step procedure that can extract all phenolic compounds. Solvent extracts may also contain some non-phenolic compounds, including caffeine. As a result, reduction of caffeine may be necessary in order to avoid criticism of green tea extracts as promoting over-consumption of caffeine. Additionally, long extraction times and high temperatures cause the degradation and epimerization of catechins which reduces the antioxidant activity of green tea extracts [13,14]. The conventional extraction methods, such as shaking and solvent extraction, have shown low efficiency and potential environmental pollution due to the large volumes of organic solvents used, as well as long extraction time and high temperature required in those methods. At present, there is interest in developing a new extraction method based on the use of environmentally friendly processes offering short extraction times, low temperatures, and small amounts of organic solvents. Supercritical fluid, microwave, and ultrasonic extraction methods are emerging as good alternatives to conventional extraction methods, mainly due to lack of need for organic solvents and relatively short extraction times [15,16,17,18]. However, the supercritical fluid extraction method has some defects such as longer extraction time and high extraction pressure, resulting in high operating costs and limiting its large-scale industrial application. The microwave extraction method removes other taste- and aroma-causing compounds from tea. One the other hand, ultrasonic extraction has been widely used in the extraction of various phenolic compounds due to the relatively low cost and simple instruments needed [19,20,21,22]. In this study, ultrasonic extraction was used to extract the green tea extracts.

Response Surface Methodology (RSM) has been widely used for the optimization of extraction conditions such as temperature, extraction time and concentration of solvents. RSM consists of mathematical and statistical techniques used to develop an adequate functional relationship between a response of interest and some independent variable.

The aim of this study was to optimize, using RSM, the ultrasonic extraction conditions for antioxidants with relatively low caffeine content from green tea. Additionally, the most important factors contributing to the antioxidant activity of green tea extracts were determined.

## 2. Results and Discussion

### 2.1. Fitting the Models

An optimization of extraction conditions for the extraction of antioxidants with relatively low caffeine content from green tea was conducted using RSM. The extraction efficiency of antioxidant compounds was influenced by extraction conditions including extraction solvent properties, extraction time, and extraction temperature [23,24]. RSM is accepted as a powerful tool in optimizing experimental conditions to maximize various responses [25,26,27,28].

For RSM, the levels of independent variables for the extraction of the antioxidant catechins were selected based on the results obtained from our preliminary experiments. The experimental design and corresponding response data are presented in Table 1. Twenty experiments were designated and six were zero point tests performed to estimate the errors. The ranges of ethanol concentration (19.7%–80.3%), extraction time (26.4–93.6 min), and extraction temperature (14.8–65.2 °C) were used. Antioxidant activity and caffeine were used as responses in the RSM experimental design. Predicted response Y for extraction of the antioxidant green tea extracts could be obtained by applying multiple regression analysis on the experimental data. The predicted quadratic polynomial models are shown in Table 2. The models were checked using a numerical method including the coefficient of determination (R^2^). R^2^ provided a measure of how well future outcomes are likely to be predicted by the model. In the models, X_1_^2^, X_2_^2^, and X_1_X_2_ were associated with synergistic effects on the antioxidant activity whereas X_1_ and X_2_ were associated with antagonistic effects. In addition, X_1_ and X_3_^2^ were associated with synergistic effects on the caffeine whereas X_1_^2^, X_2_^2^, and X_1_X_3_ were associated with antagonistic effects. The R^2^ of the models for antioxidant activity and caffeine was 0.8961 and 0.9101, respectively. Moreover, the coefficient of variation (CV) was 2.27 and 3.68, respectively, which indicates that a relatively lower value of CV showed a better reliability of the response model.

In general, lack of fit test for the model describes the variation in the data around the fitted model [29]. If the model does not fit the data well, the value of lack of fit will be significant and then proceeding with investigation and optimization of the fitted response surface is likely to give misleading results. Table 3 shows the analysis of variance of the fitted quadratic polynomial model for antioxidant activity and caffeine. For antioxidant activity, the linear parameters (X_1_, X_2_) and interaction parameters (X_1_X_2_, X_1_X_3_) were significant at the level of *p* < 0.01 and quadratic parameter X_1_^2^ was significant at the level of *p* < 0.05. In the caffeine, X_1_ and X_1_X_2_ were significant at the level of *p* < 0.001 and X_2_X_3_ was significant at the level of *p* < 0.05. Moreover, the models used to fit response variable were significant (*p* < 0.01) and the lack of fit was not significant (*p* > 0.05) for all responses. It is indicated that the models used to fit responses variable were adequate to represent the relationship between the response values and the independent variables.

### 2.2. Response Surface Optimization of Ultrasonic Extraction

To visualize the relationship between the response and experimental levels of the independent variables for the antioxidants extraction, three-dimensional (3D) surface plots were constructed according to the quadratic polynomial model equations of Table 2.

**Table 1:** Coded and processed variables levels used in experimental design for RSM.

Run No.	Coded and Processed Variable Level			Response (mg/g)	
	X_1_	X_2_	X_3_	Y_1_	Y_2_
	Ethanol concentration (%)	Extraction time (min)	Extraction temperature (°C)	Antioxidant activity (%)	Caffeine content (mg/g)
1	0 (50)	0 (60)	1.682 (65.2)	80.48	19.49
2	0 (50)	0 (60)	0 (40)	79.77	18.54
3	−1 (32)	−1 (40)	−1 (25)	79.43	16.84
4	1 (68)	−1 (40)	1 (55)	80.22	18.08
5	0 (50)	0 (60)	0 (40)	79.58	17.96
6	0 (50)	0 (60)	0 (40)	79.51	18.22
7	0 (50)	−1.682 (26.4)	0 (40)	79.49	17.93
8	0 (50)	0 (60)	0 (40)	79.09	18.07
9	−1 (32)	1 (80)	−1 (25)	79.62	17.31
10	1 (68)	−1 (40)	−1 (25)	79.62	18.04
11	0 (50)	0 (60)	0 (40)	78.87	18.00
12	−1.682 (19.7)	0 (60)	0 (40)	79.67	16.39
13	1 (68)	1 (80)	−1 (25)	82.99	18.33
14	0 (50)	0 (60)	0 (40)	77.28	18.56
15	0 (50)	0 (60)	−1.682 (14.8)	77.07	18.08
16	0 (50)	1.682 (93.6)	0 (40)	82.36	18.04
17	1.682 (80.3)	0 (60)	0 (40)	83.78	18.01
18	−1 (32)	−1 (40)	1 (55)	80.84	17.33
19	1 (68)	1 (80)	1 (55)	83.6	18.67
20	−1 (32)	1 (80)	1 (55)	81.45	18.09

**Table 2:** Coded and processed variables levels used in experimental design for RSM.

Responses	Quadratic polynomial model equations	R^2^	CV(%)
Antioxidant activity	91.5074 *** − 0.3590X_1_ ** − 0.2940X_2_ ** + 0.0857X_3_ + 0.0032X_1_^2^ ** + 0.0019X_2_^2^ ** + 0.0000X_3_^2^ + 0.0021X_1_X_2_* − 0.0009X_1_X_3_ + 0.0002X_2_X_3_	0.8961	2.27
Caffeine	12.1785 *** + 0.1707X_1_ *** + 0.0394X_2_ − 0.0341X_3_ − 0.0012X_1_^2^ *** − 0.0003X_2_^2^ + 0.0007X_3_^2^ * − 0.0001X_1_X_2_ − 0.0004X_1_X_3_ * + 0.0002X_2_X_3_	0.9101	3.68
content			

CV, coefficient of variation; X_1_, ethanol concentration; X_2_, extraction time; X_3_, extraction temperature. * Significant at *p* < 0.05; ** Significant at *p* < 0.01; *** Significant at *p* < 0.001.

**Figure 1:**
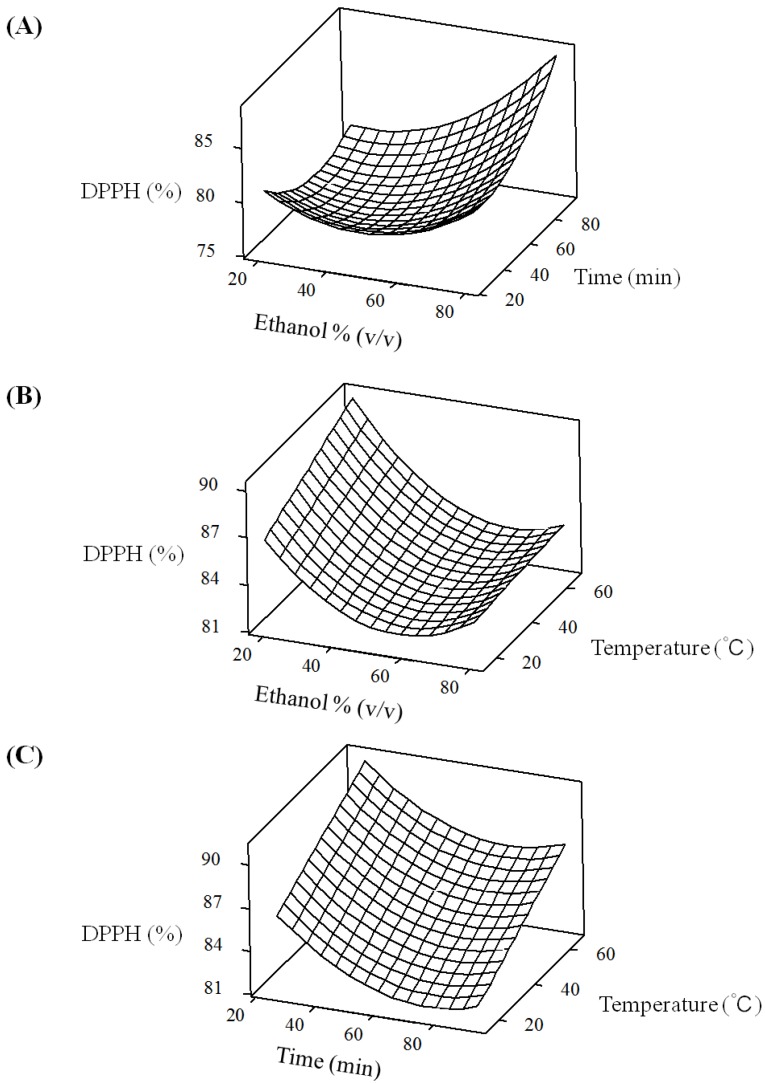
Response surface plots for the effects of ethanol concentration, extraction time and temperature on antioxidant activity of green tea extracts. (**A**) Ethanol concentration and extraction time; (**B**) ethanol concentration and extraction temperature; (**C**) extraction time and temperature.

**Figure 2:**
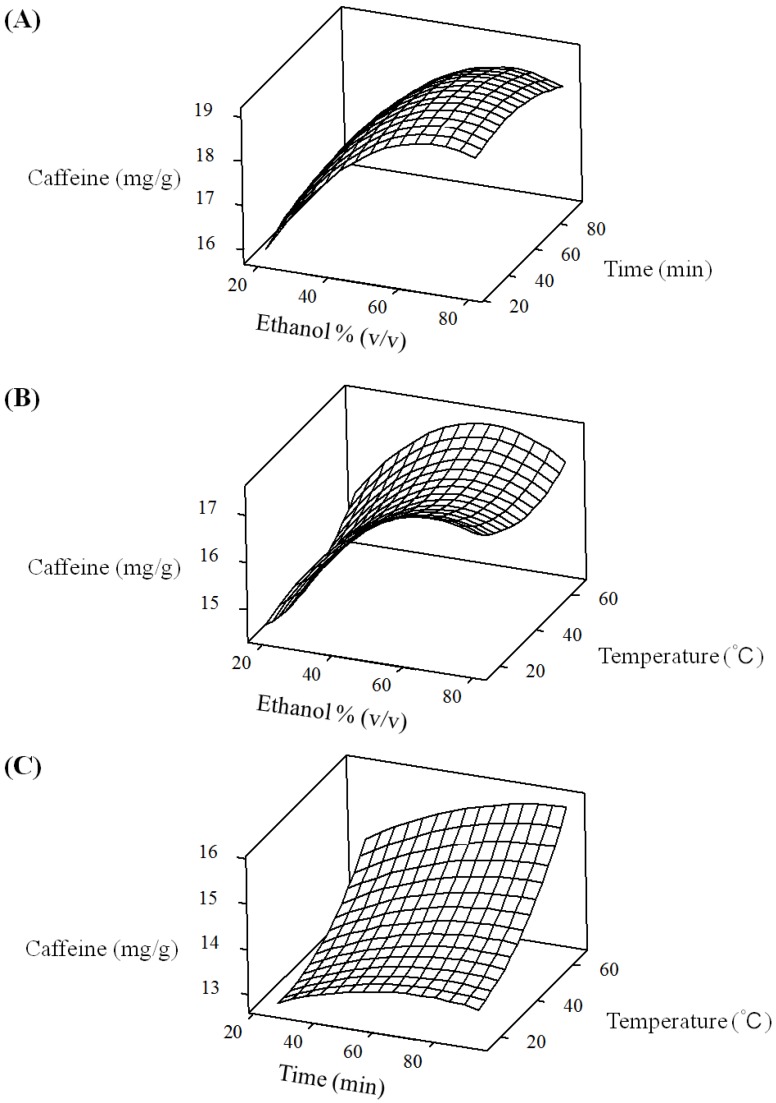
Response surface plots for the effects of ethanol concentration, extraction time and temperature on caffeine contents of green tea extracts. (**A**) Ethanol concentration and extraction time; (**B**) ethanol concentration and extraction temperature; and (**C**) extraction time and temperature.

The effect of the variables and their interaction on the responses can be seen Figure 1 and Figure 2. As shown in Figure 1A, when extraction temperature was fixed at 0 level, antioxidant activity was increased slightly by increasing ethanol concentration from 37% to 80.3% and reached the maximum value at the highest ethanol concentration in the fixed extraction time of 60 min. Figure 1B shows the effect of the interaction of ethanol concentration and extraction temperature on the antioxidant activity at a fixed extraction time of 0 level. Maximum antioxidant activity was obtained at the lowest ethanol concentration and then decreased slightly by increasing ethanol concentration to 60% in the fixed extraction temperature of 40 °C. Figure 1C shows the effect of the interaction of extraction time and extraction temperature on the antioxidant activity at a fixed ethanol concentration of 0 level. Maximum antioxidant activity was obtained at the lowest extraction time and then decreased slightly by increasing extraction time in the fixed extraction temperature of 40 °C.

**Table 3:** Analysis of variance results for the regression equation.

Source	Degree of freedom	Sum of squares	Mean square	*f*-value	*p*-value
**Antioxidant activity**					
Model	9	56.6980	5.9372	9.59	0.001
X_1_ (Ethanol%, v/v)	1	10.5842	9.8906	15.05	0.003
X_2_ (Time, min)	1	11.2031	7.7975	11.87	0.006
X_3_ (Temperature, °C)	1	7.5784	0.3988	0.61	0.454
X_1_X_2_	1	13.9271	15.6784	23.86	0.001
X_1_X_3_	1	8.4326	8.3531	12.71	0.005
X_2_X_3_	1	0.0000	0.0000	0.00	0.996
X_1_^2^	1	4.4342	4.4342	6.75	0.027
X_2_^2^	1	0.5157	0.5157	0.78	0.396
X_3_^2^	1	0.0226	0.0226	0.03	0.857
Lack of fit	5	2.4163	0.4833	0.58	0.717
**Caffeine content**					
Model	9	7.5879	0.8431	11.24	0.000
X_1_ (Ethanol%, v/v)	1	2.8716	2.2364	29.82	0.000
X_2_ (Time, min)	1	0.3873	0.1404	1.87	0.201
X_3_ (Temperature, °C)	1	1.1878	0.0633	0.84	0.380
X_1_X_2_	1	2.3152	2.2194	29.60	0.000
X_1_X_3_	1	0.2536	0.1920	2.56	0.141
X_2_X_3_	1	0.4088	0.4088	5.45	0.042
X_1_^2^	1	0.0162	0.0162	0.22	0.652
X_2_^2^	1	0.1032	0.1032	1.38	0.268
X_3_^2^	1	0.0442	0.0442	0.59	0.460
Lack of fit	5	0.3907	0.0781	1.09	0.464

Figure 2A shows the effect of the interaction of ethanol concentration and extraction time on the caffeine contents at a fixed extraction temperature of 0 level. Minimum caffeine value was obtained at the lowest ethanol concentration and reached the maximum value at 70% of ethanol concentration in the fixed extraction time of 60 min. Figure 2B shows the effect of the interaction of ethanol concentration and extraction temperature on the caffeine contents at a fixed extraction time of 0 level. Minimum caffeine value was also obtained at the lowest ethanol concentration and reached the maximum value at 78% of ethanol concentration in the fixed extraction temperature of 40 °C. Figure 2C shows the effect of the interaction of extraction time and extraction temperature on the caffeine contents at a fixed ethanol concentration of 0 level. Minimum caffeine value was obtained at the lowest extraction time and reached the maximum value at 79 min of extraction time in the fixed extraction temperature of 40 °C. Moreover, we have found that ethanol concentration (X_1_) was the most significant factor affecting the responses at the level of *p* < 0.01.

### 2.3. Optimization and Verification of the Model for Ultrasonic Extraction

Optimum process parameters achieved by maximizing antioxidant activity and simultaneously minimizing caffeine contents. During the optimization stage, the desirability function of the MINITAB (Minitab Inc., State College, PA, USA) statistical software is used to obtain the best compromise of the two responses with the weights of all 1.0. As shown in Table 4, the predicted optimal conditions for ultrasonic extraction were found at 19.7% ethanol, 26.4 min extraction time and 24.0 °C extraction temperature. In the predicted optimal conditions, the experimental yield of antioxidant activity and caffeine were 82.1% and 15.4 mg/g, respectively.

**Table 4:** Optimum conditions and the predicted and experimental value of responses at the optimum conditions.

Variables	Optimum conditions (predicted)	Modified conditions (actual)
Ethanol (%)	19.7	20.0
Extraction time (min)	26.4	26.0
Temperature (°C)	24.0	24.0
Antioxidant activity (%)	82.1	82.7 ± 0.47
Caffeine (mg/g)	15.4	15.7 ± 0.56

In order to verify the accuracy of the model for predicting maximal yield, we performed actual experiments using the optimized extraction conditions. The experimental values were very close to the predicted ones. This indicated that the optimization achieved in the present study was reliable. Horžić *et al*. reported that the best extraction performance of polyphenols and methylxanthines from yellow tea was achieved with the use of an ultrasound probe, especially in combination with 75% aqueous ethanol as an extraction medium [30]. This disagreement could be due to a difference in cultivar and/or target compounds in the extracts. In general, effective separation of antioxidants from a complex plant matrix is a difficult procedure due to degradation of antioxidants and co-extraction of other various compounds, which are undesirable in the antioxidant extract. High extraction temperatures can increase the yield of tea catechins because the cell walls of the green tea leaves become more permeable to the solvent and to the constituents [31,32]. However, the catechins can also be subject to degradation and epimerization when the extraction is conducted at too high temperatures [14]. This epimerization is undesirable because a large amount of the most important catechin, EGCg, is transformed into GCg [33]. Conversely, extraction at low temperatures is desirable to avoid these changes, while the efficiency of the extraction is low. Thus, it was necessary to add some organic solvent to the water to improve the efficiency of extraction of catechins from green tea. The most widely used solvents for extracting phenolic compounds are methanol, ethanol, acetone, and their water mixtures [34,35,36,37,38]. Especially, ethanol and water mixtures are commonly used for the extraction of phenols from plant materials. This is due to the wide range of phenols that the aqueous ethanol mixtures can dissolve and ethanol mixtures are acceptable for human consumption models [38].

### 2.4. Comparison with Conventional Extraction

In order to compare extraction efficiency, ultrasonic extraction was performed with the optimum conditions by RSM and conventional shaking extraction was carried out according to modified conditions reported by some of the previous and preliminary studies. Different reports are found in the literature. Gramza *et al*. reported that the antioxidant activity of green tea extracts was higher in 95% ethanol extracts than that of hot water extracts [39]. Grujic *et al*. reported that the antioxidant activity of Mate tea extracts was higher in 40% ethanol extracts than that of 50% and 60% ethanol concentration, respectively [40]. Mohammedi *et al*. reported that the antioxidant activity of *Tamarix aphylla* leaves extracts was higher in 70% ethanol extracts than that of water extracts [41]. Chew *et al*. reported that the antioxidant activity of *Centella asiatica* tea extracts was higher in 60% ethanol extracts than that of other concentrations of ethanol [42]. Xu *et al*. reported that the optimal conditions for phenolic antioxidants with shaking extraction from tea fruit peel were 43% ethanol, 33 min extraction time and 60 °C extraction temperature [43]. In preliminary study, we found that the high content of phenolic compounds ranged from 50% to 80% ethanol concentration in the green tea (data not shown). This disagreement could be connected with a polarity of extracts.

In general, solvents extract those phytochemicals which have similar polarity as the solvents. Although the relationship between the molecular structure and their antioxidant activity were not investigated in this study, the literature suggests that the antioxidant activity of plant extracts is strongly dependent on the solvent due to the different antioxidant potentials of compounds with different polarity. Also, it should be taken into consideration that a higher ethanol to water ratio had a positive influence on the antioxidant activity. Thus, we determined with 70% ethanol, 120 min extraction time and 30 °C extraction temperature the reference method in comparison to the ultrasonic extraction.

The ethanol concentration and extraction time used for antioxidant extraction were significantly reduced by 72% and 78% respectively, compared to the conventional extraction. The contents of caffeine were also lowered 30% compared to the conventional extraction. However, antioxidant activity was not found to be significantly different in the different extraction methods. These results could be very useful when attempting to reduce the caffeine content in green tea. This result was supported by the Saito *et al*., who studied a comparison of EGCG/caffeine ratios according to the extraction system used on Brazilian green tea [44]. It is described that EGCG/Caffeine level was higher in ultrasound-assisted extraction by immersed ultrasonic bath than that in water extraction by heated magnetic stirrer. Tang also reported that ultrasonic-enhanced supercritical fluid extraction was effective at removing caffeine from green tea without damaging the structure of its active components [45]. However, this technique has some defects such as long extraction time (4 h) and high extraction pressure (30 MPa), resulting in high operating costs and limiting its large-scale industrial application.

Therefore, ultrasonic extraction is a promising extraction technique for antioxidants with relatively lower level caffeine from green tea, which significantly reduced the solvent usage and extraction time compared to conventional shaking extraction.

### 2.5. Correlations between Antioxidant Activity and Catechins Composition

Green tea contains various classes of polyphenols, and up to 90% of these polyphenols is composed of catechins. To determine the most important factors affecting the antioxidant activity, Pearson’s correlation coefficients were calculated using the experimental RSM data. Antioxidant activity and related compounds including total polyphenol, total catechins, gallated catechins, and ratio of EGCg + ECg/EGC were used as factors (Table 5). Correlations between factor values and the antioxidant activity obtained from RSM are shown in Table 6. In the present study, the ratio of EGCg + ECg/EGC values had the highest correlations to antioxidant activity (r = 0.491, *p* = 0.028). The ratio of EGCg + ECg/EGC was worth considering since the three catechins, EGCg, ECg, and EGC, represent about 80% of the total catechins in green tea. In general, EGCg and ECg are usually accepted as quality indicators of antioxidant activity in tea products [14]. This result supports that antioxidant activity relates not only to the amounts of antioxidants but also to the properties of antioxidants such as chemical structure and interactions among each other [46]. Generally, the antioxidant capacity has often been correlated with the phenolic content [47,48,49]. However, some authors have reported that the antioxidant activity was not correlated with the phenolic content [50,51]. This disagreement suggested that antioxidant activity was strongly dependent on the solvent due to the different antioxidant potentials of compounds with different polarity [52,53].

**Table 5:** Response values-related antioxidants obtained from experimental design.

Run No.	Total polyphenol	Total catechins	Gallated catechins	(EGCg + ECg)/EGC
1	167.06	123.36	57.51	1.22
2	160.70	115.09	51.92	1.15
3	141.43	91.33	34.61	0.85
4	153.94	122.14	60.13	1.36
5	157.79	112.13	50.73	1.15
6	155.70	112.96	50.97	1.15
7	150.62	112.60	51.24	1.17
8	154.61	113.20	51.43	1.16
9	142.71	92.37	34.29	0.83
10	147.65	123.99	61.87	1.39
11	159.89	112.77	51.35	1.17
12	139.53	84.21	29.81	0.77
13	153.19	126.29	63.62	1.42
14	156.91	115.70	52.78	1.17
15	148.48	104.56	45.05	1.05
16	155.70	113.09	51.50	1.17
17	144.94	130.66	68.91	1.57
18	150.31	99.19	40.80	0.98
19	157.45	134.51	69.23	1.47
20	151.30	102.78	41.97	0.96

Total catechins: sum of EGC, EC, GC, C, EGCg, ECg and GCg; Gallated catechins: sum of EGCg, ECg and GCg.

**Table 6:** Correlations between the antioxidant activity and response values obtained from the experimental design.

Responses	Pearson’s correlation coefficient	*p*-value
Total polyphenol	0.015	0.951
Total catechins	0.461	0.041
Gallated catechins	0.486	0.030
(EGCg + ECg)/EGC	0.491	0.028

Total catechins: sum of EGC, EC, GC, C, EGCg, ECg and GCg; Gallated catechins: sum of EGCg, ECg and GCg.

## 3. Experimental

### 3.1. Tea Materials and Chemicals

Green tea leaves (*Camellia sinensis* O. Kuntz) were purchased from Amorepacific Co., Ltd. (Seoul, Korea) and were pulverized to mean particle size of 20 µm by jet-milling. Folin–Ciocalteu’s phenol reagent, gallic acid, caffeine, (+)-catechin, (−)-epicatechin, (−)-epicatechin gallate, (+)-catechin gallate, (−)-epigallocatechin, (+)-gallocatechin, (−)-epigallocatechin gallate, (+)-gallocatechin gallate, ascorbic acid, and 2,2-diphenyl-1-picrylhydrazil (DPPH) were purchased from Sigma-Aldrich Co. All the reagents used in the HPLC analyses were of HPLC grade.

### 3.2. Ultrasonic Extraction

The ultrasonic extraction was treated with an ultrasonic probe (SEE-SONIC II, UL-Tech. Com., Uiwang, Korea) with an ultrasonic power of 150 watts and a frequency of 20 kHz controlled with a phase-locked loop system. The probe had a titanium horn of 2.5 cm in diameter and irradiated with an ultrasonic wave directly from the horn. Five g of green tea powder was mixed with the appropriate solvent (500 mL) in the stainless steel vessel and extracted by ultrasonic probe, which was immersed in the vessel. The resulting suspensions were centrifuged at 15,000 g for 10 min and filtered by a 0.45 µm syringe filter (CE Minisart RC 15, Sartorius, Goettingen, Germany) before further analysis.

### 3.3. Conventional Extraction

Shaking extraction, one of conventional extraction methods, was performed with 5 g green tea powder mixed with 500 mL of 70% ethanol concentration and extracted at 30 °C for 120 min in shaking incubator. The resulting suspensions were centrifuged at 15,000 g for 10 min and filtered by a 0.45 µm syringe filter (CE Minisart RC 15) before further analysis.

### 3.4. Experimental Design and Statistical Analysis

Ultrasonic extraction optimized the experimental design using RSM. A Central Composite Design (CCD) consisting of twenty experimental runs was employed including six star points (α = 1.682) points, eight factorial points and six central points. The independent variables were the ethanol concentration (X_1_), extraction time (X_2_), and extraction temperature (X_3_) while dependent variables (response) were antioxidant activity (Y_1_), and caffeine contents (Y_2_). The range values of the three independent variables were determined by preliminary study. Experiments were performed in replicate and the average values were used as the response, Y. A full polynomial model was obtained with a multiple regression technique for three factors using MINITAB 16 (Minitab Inc., State College, PA, USA).

### 3.5. Measurement of Antioxidant Activity

The antioxidant activity of green tea extracts was measured using a DPPH free radical scavenging assay according to the modified method described by Yamaguchi *et al*. [54]. The green tea extracts (0.5 mL) were diluted 10-fold with extract solvent and mixed with 0.12 mM DPPH solution (2.5 mL). The mixture was vortex mixed and allowed to stand at room temperature for 30 min. The absorbance of the resulting solution was measured at 517 nm and the scavenging activity of DPPH free radicals was calculated by using the following formula: scavenging activity (%) = 100 − [(absorbance of sample at 517 nm/absorbance of blank at 517 nm) × 100]. Blank is the absorbance of the control reaction which contains all of the components without the tested sample.

### 3.6. Measurement of Caffeine and Catechins Contents

**Figure 3:**
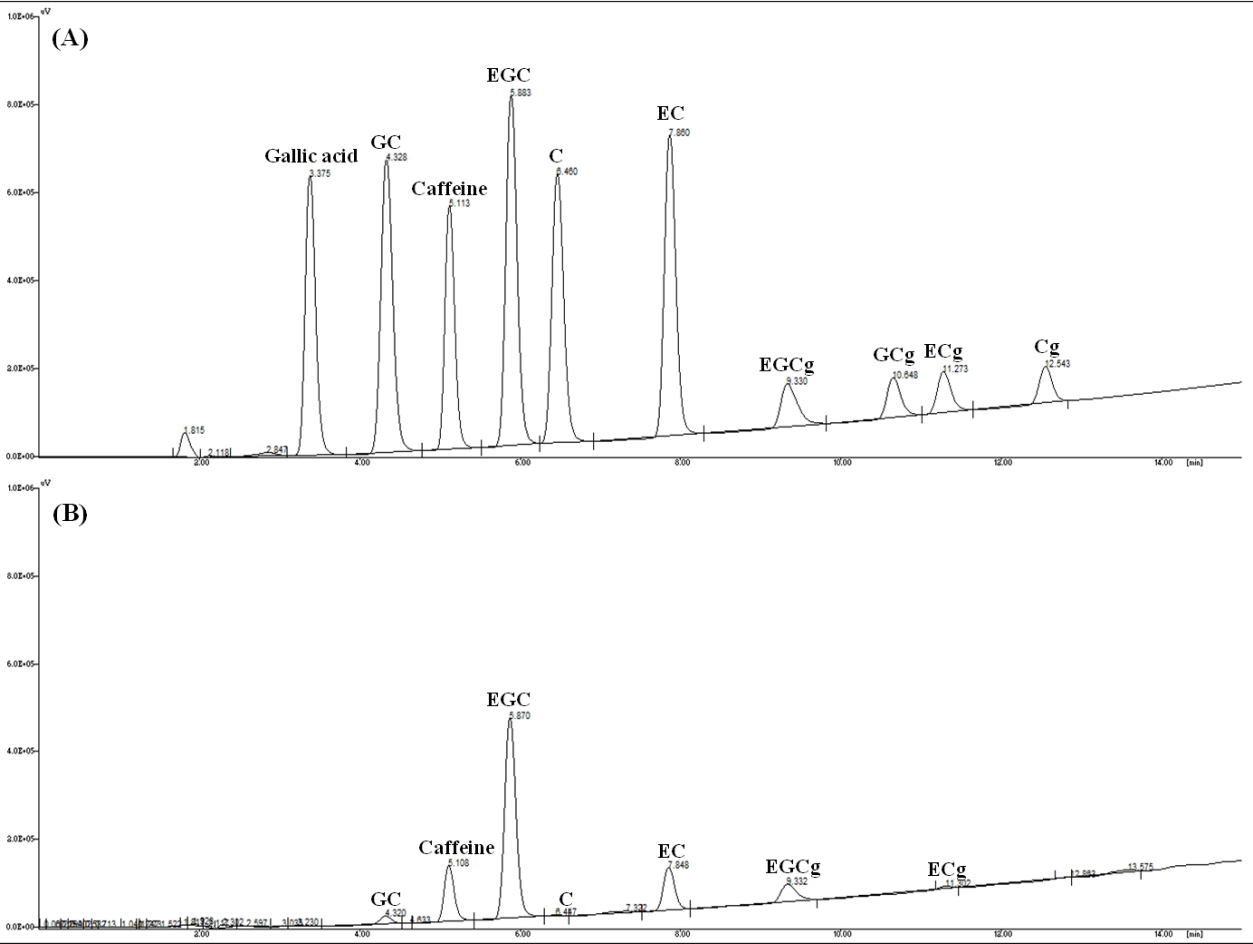
HPLC chromatograms of standard solution of caffeine and catechins (**A**) and extracts (**B**). GC, (+)-gallocatechin; EGC, (−)-epigallocatechin; EC, (−)-epicatechin; EGCg, (−)-epigallocatechin gallate; GCg, (+)-gallocatechin gallate; ECg, (−)-epicatechin gallate; Cg, (+)-catechin gallate.

The contents of caffeine and catechins in the green tea extracts were analyzed according to the modified method described by Hu *et al*. [55]. Prepared sample analyzed by HPLC (JASCO Co., Tokyo, Japan) using a XTerra RP18 column (3.5 µm, 4.6 × 150 mm, Waters, Milford, MA, USA) at 40 °C and multi-wavelength detector (MD-2010 Plus, JASCO Co., Tokyo, Japan) was set at 210 nm (Figure 3). The mobile phase was composed of two solution A (0.2% ortho phosphoric acid) and solution B (methanol) and eluted with a linear gradient elution of 0 min, 82% A; 15 min, 40% at a flow rate of 1.0 mL/min. Individual catechins and caffeine contents were calculated by comparing with an external standard calibration curves of each analyte. All the analytes showed good linearity (R^2^ > 0.99) in a relatively wide concentration range.

### 3.7. Measurement of Total Polyphenol Contents

The contents of total polyphenol in the green tea extracts were measured by the modified Folin-Ciocalteu assay carried out according to the method described by Singleton and Rossi [56]. The extracts (0.1 mL) were diluted 10-fold with extract solvent and mixed with 0.2 N Folin-Ciocalteu reagent (1 mL). The mixture was allowed to stand at room temperature for 3 min after which 1 mL of saturated sodium carbonate was added. The final mixture was incubated at room temperature for 60 min and absorbance of the resulting solution measured at 735 nm. The contents of total polyphenol were calculated by comparing with an external standard calibration curve of gallic acid (R^2^ = 0.9983) and were expressed as gallic acid equivalents (GAE, g gallic acid) per 100 g of sample

## 4. Conclusions

In the present study, response surface methodology was used to optimize the ultrasonic extraction of phenolic antioxidants with relatively low caffeine content from green tea. A central composite design was used to determine the optimum process parameters and the second order polynomial models for predicting responses were obtained. Ethanol concentration was the most significant factor affecting antioxidant activity and caffeine content and the optimal extraction conditions were 19.7% ethanol for 26.4 min at 24.0 °C. Under optimized conditions the experimental values were very close to the predicted values. Compared to the conventional shaking extraction methods, ultrasonic extraction requires less extraction time, lower temperature and provides lower caffeine content without a decrease of antioxidant activity. As such, it may be said that ultrasonic extraction is an effective and practical method for obtaining phenolic antioxidants with relatively low caffeine content from green.
